# A dual timeline of safinamide effects in Parkinson’s disease: mechanistic rationale and clinical implications

**DOI:** 10.3389/fneur.2026.1829241

**Published:** 2026-06-03

**Authors:** Roongroj Bhidayasiri, Thanarat Suansanae

**Affiliations:** 1 Chulalongkorn Centre of Excellence for Parkinson’s Disease and Related Disorders, Department of Medicine, Faculty of Medicine, Chulalongkorn University and King Chulalongkorn Memorial Hospital, Thai Red Cross Society, Bangkok, Thailand; 2 The Academy of Science, The Royal Society of Thailand, Bangkok, Thailand; 3 Department of Pharmacy, Faculty of Pharmacy, Mahidol University, Bangkok, Thailand

**Keywords:** dual mechanism, dyskinesia, monoamine oxidase type B inhibitor, motor benefits, Parkinson’s disease, safinamide, glutamatergic transmission

## Abstract

Safinamide is a selective, reversible monoamine oxidase type B inhibitor (MAO-B inhibitor) used as an adjunct to oral levodopa in patients with Parkinson’s disease (PD) experiencing motor fluctuations. Unlike other MAO-B inhibitors, safinamide possesses a dual mechanism of action, combining dopaminergic enhancement through MAO-B inhibition with modulation of abnormal glutamatergic transmission. In clinical practice, safinamide is primarily recognized for its ability to reduce motor fluctuations. However, its pharmacological profile suggests a broader therapeutic potential beyond dopaminergic modulation alone. Randomized controlled trials have shown that safinamide increases daily ON time without worsening troublesome dyskinesia. Nevertheless, reported effects on dyskinesia have generally been modest and inconsistent across studies. Clinical observations from longer-term follow-up and real-world studies suggest that improvements in dyskinesia and certain non-motor symptoms may emerge more gradually than the early motor benefits. Based on this observed pattern, it can be hypothesized that safinamide may exhibit a dual timeline of clinical effects, with early dopaminergic improvements in motor ON time followed by later modulation of dyskinesia and selected non-motor symptoms potentially mediated through glutamatergic mechanisms. On the basis of these pharmacological characteristics and clinical observations, a phenotype-oriented approach to patient selection for safinamide therapy may be considered. In addition, recognizing the potential for a dual timeline of therapeutic effects may help clinicians guide patients’ expectations of treatment and interpret treatment response, as well as optimizing follow-up strategies in routine clinical practice.

## 1 Introduction

Oral levodopa is recognized as the mainstay of the initial therapy for the management of the early stage of Parkinson’s disease (PD) providing effective control of motor symptoms (1). However, over time with chronic treatment, the usual dose of levodopa often has increasingly less clinical benefit and as a result patients can experience disabling motor complications (motor fluctuations and dyskinesias) that impact their functional ability and quality of life (2–5). In clinical practice, when optimization of levodopa therapy (for example, through adjustments in dose or dosing frequency) fails to provide satisfactory control of motor complications, adjunctive treatment is typically introduced (6). Available options include dopamine agonists, catechol-O-methyltransferase (COMT) inhibitors, and monoamine oxidase type B (MAO-B) inhibitors. These agents are added to enhance and prolong the therapeutic effects of levodopa, thereby improving the consistency of motor response and overall symptom control.

Safinamide is a MAO-B inhibitor approved as an add-on therapy for patients with PD experiencing motor fluctuations (7). In routine clinical practice, when clinicians are considering adjunctive treatments, it is often grouped with other MAO-B inhibitors, and its use is commonly confined to this therapeutic category. However, safinamide differs pharmacologically from first-generation MAO-B inhibitors in that it combines selective, reversible MAO-B inhibition with modulation of abnormal glutamatergic transmission (8, 9). While its clinical efficacy in reducing daily motor OFF-time has been demonstrated in controlled studies (10–12), the implications of its dual mechanism for treatment positioning across different disease stages and clinical phenotypes remains an area of active discussion. Current treatment algorithms primarily emphasize its role in managing motor fluctuations, yet its pharmacological profile suggests potential relevance beyond a purely dopaminergic framework. The dual mechanism of safinamide raises the possibility that its dopaminergic and non-dopaminergic effects may emerge over different time scales.

**Figure 1:**
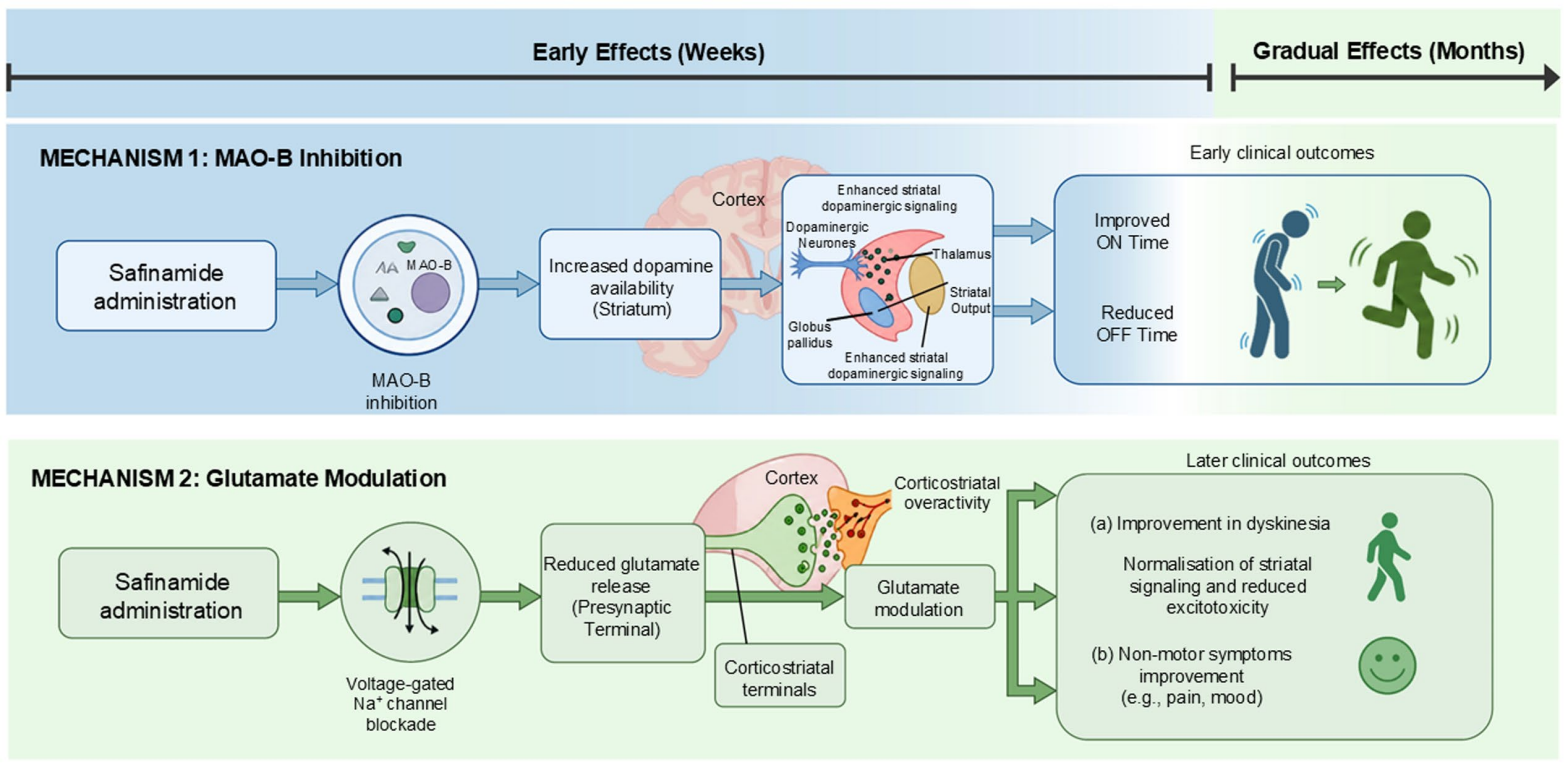
Conceptual framework for the proposed dual timeline of safinamide effects in Parkinson’s disease. Safinamide may exert temporally distinct pharmacological effects through two complementary mechanisms. Early reversible MAO-B inhibition increases striatal dopamine availability and enhances dopaminergic signalling, contributing to improvements in motor fluctuations, including increased ON time and reduced OFF time. In contrast, modulation of glutamatergic transmission through voltage-gated sodium channel blockade and reduced stimulated glutamate release may gradually influence corticostriatal overactivity and basal ganglia network dynamics, potentially contributing to later changes in dyskinesia and selected non-motor symptoms. The timeline is schematic and does not imply proportionality between early and gradual effects. The extended arrow in the gradual-effects phase indicates that these later modulatory effects, if present, are expected to evolve and potentially continue over time. This model remains hypothesis-generating and should not be interpreted as evidence of a proven causal sequence.

Here we review the mechanistic rationale underlying safinamide’s dual mechanism of action, summarize the available clinical evidence, and explore how it may be positioned within a phenotype-oriented approach to PD management. We hypothesize that the clinical effects of safinamide in PD may follow a dual temporal pattern driven by its distinct pharmacological mechanisms. Early therapeutic benefits are primarily mediated by potent MAO-B inhibition and enhanced dopaminergic transmission, whereas modulation of glutamatergic pathways may contribute to more gradual effects on dyskinesia and certain non-motor symptoms (NMSs) (Figure 1). Rather than proposing definitive changes to current practice, our aim is to encourage reflection on whether a mechanism-informed strategy might help refine therapeutic choice and sequencing in selected patient populations. By integrating pharmacological principles with clinical observations, this article seeks to provide a structured framework for discussion and to identify areas where further research may clarify the optimal role of safinamide in contemporary PD care.

This ‘Hypothesis and Theory’ article should be interpreted as a narrative, hypothesis-generating synthesis rather than a systematic review or meta-analysis. Relevant literature was selected to support development of the proposed conceptual model, with emphasis on pivotal randomized controlled trials (RCTs) of safinamide, post-hoc and subgroup analyses, observational real-world studies, and mechanistic pharmacological or pharmacokinetic–pharmacodynamic studies addressing MAO-B inhibition, glutamatergic modulation, motor fluctuations, dyskinesia, and non-motor symptoms. Reference lists of key publications were also reviewed to identify additional relevant sources. No formal systematic search strategy, study quality scoring, or meta-analysis was undertaken.

In the context of this article, we have used the term ‘dual timeline’ to describe the proposed temporal dissociation between early dopaminergic clinical effects and later, potentially glutamatergic-mediated modulatory effects. This terminology is intended to denote a hypothesis-generating clinical framework rather than a proven biphasic pharmacological response.

## 2 Pharmacological basis for a dual dopaminergic–glutamatergic timeline

Safinamide is a selective and reversible inhibitor of MAO-B with additional non-dopaminergic properties that modulate glutamatergic transmission. This dual mechanism distinguishes safinamide from other MAO-B inhibitors and provides a potential pharmacological basis for its clinical profile in PD (13). The primary pharmacodynamic effect of safinamide is potent MAO-B inhibition, which enhances synaptic dopamine availability by reducing enzymatic dopamine degradation within the striatum. *In vitro* studies demonstrate MAO-B inhibition at nanomolar concentrations (IC₅₀ ≈ 0.098 μM), and pharmacokinetic–pharmacodynamic studies in humans have shown dose-dependent and near-complete MAO-B inhibition at relatively modest oral doses (8). Through this mechanism, safinamide enhances dopaminergic signaling and contributes to improvements in motor fluctuations observed in RCTs (14, 15).

In addition to MAO-B inhibition, safinamide modulates glutamatergic transmission through state-dependent blockade of voltage-gated sodium channels and reduction of excessive glutamate release (16, 17). Experimental studies indicate that these antiglutamatergic effects occur at concentrations in the micromolar range, substantially higher than those required for MAO-B inhibition. Electrophysiological recordings from striatal networks show that safinamide produces dose-dependent reductions in stimulated glutamate release at concentrations of approximately 3–5 μM, which lie near the upper range of therapeutic brain concentrations achieved during clinical treatment (17). This observation supports the notion of an approximate two-order-of-magnitude difference in potency between the drug’s dopaminergic and antiglutamatergic mechanisms. The requirement for higher concentrations to engage glutamatergic mechanisms compared with MAO-B inhibition provides a plausible pharmacodynamic explanation for the temporal dissociation between early dopaminergic motor effects and later modulatory effects on dyskinesia and selected NMSs.

From a systems perspective, excessive glutamatergic activity within basal ganglia circuits has been implicated in the pathophysiology of levodopa-induced dyskinesia (18). By reducing abnormal glutamate release and stabilizing neuronal excitability, safinamide may contribute to modulation of these maladaptive network processes over time (19). Together, these pharmacological properties provide a biologically plausible framework for the dual-timeline hypothesis proposed in this article, in which early therapeutic benefits are primarily mediated through enhanced dopaminergic transmission while slower modulation of glutamatergic pathways may contribute to longer-term effects on dyskinesia and motor circuit dynamics.

## 3 Safinamide and motor PD symptoms: early versus later effects

Evidence for the efficacy and safety profile of safinamide has been gathered from RCTs, accompanied in some cases by post-hoc subgroup analyses, and supported by data from observational real-world studies.

### 3.1 Randomized, controlled trials

Multiple Phase III pivotal RCTs have reported that safinamide (at doses of 50 or 100 mg daily) as an add-on to levodopa in PD patients can significantly increase daily ON time with no or non-troublesome dyskinesia, decrease daily OFF time, improve overall motor function as well as having beneficial effects on quality of life (14, 15, 20, 21). In these studies, the reported improvement in daily motor ON-time following safinamide treatment occurred rapidly, within 1–2 weeks of starting treatment due to potent, early-onset MAO-B inhibition which blocks dopamine breakdown, thereby enhancing the effects of levodopa. However, to date, reports of the effects of safinamide on dyskinesia, particularly in the early stages of treatment, are somewhat equivocal and while the therapeutic effects of safinamide on ON-time have been observed without worsening of levodopa-induced dyskinesia (22), they are modest, and whether it has specific antidyskinetic effects remain to be confirmed. The absence of worsening dyskinesia in clinical trials does not necessarily imply a direct antidyskinetic effect, but rather suggests that increased ON-time may occur without exacerbating dyskinesia in many patients. Looking at the longer term treatment effect of safinamide, pivotal study 018 found that the change in Dyskinesia Rating Scale (DRS) score was not significantly different between the safinamide and placebo groups after 24 months of treatment (14).

### 3.2 Post-hoc and subgroup analyses

Several post-hoc and subgroup analyses of the pivotal safinamide trial data have been performed, and these can provide additional insights into the effects of safinamide as an adjunctive PD therapy to levodopa in specific subgroups of patients.

An analysis of data from Study 018 for a subgroup of patients who had moderate-to-severe dyskinesia at baseline (a DRS score of >4; 36% of the total cohort) found a significant decrease in DRS scores with safinamide at a dose of 100 mg/day compared with placebo at 24 months (*p* = 0.0317) (14). In addition, a *post hoc* analysis the Japanese patient cohort found that while overall safinamide did not cause marked dyskinesia, in patients with pre-existing dyskinesia at baseline, ON-time with troublesome dyskinesia showed a transient increase up to Week 4 but then gradually decreased up to Week 52 and the incidence of new-onset dyskinesia was low (23).

A post-hoc analyses of combined data from the RCTs Study 016 and the SETTLE study (a pooled dataset of 971 patients) evaluated the effect of safinamide in two subgroups of patients: those who were receiving levodopa only at baseline versus those receiving multiple concomitant PD therapies (dopamine agonists and/or COMT-I, with or without amantadine) (24). The results showed that ON-time with no or non-troublesome dyskinesia was improved with safinamide treatment versus placebo regardless of the number of other concomitant PD medications being taken.

A further post-hoc analysis of data from the SETTLE study stratified patients according to their response to safinamide treatment (early, late, transient or poor) and found that in patients who showed a significant early treatment response, the response was more likely to be sustained for a longer period of time than in other responder subgroups (25). Possible differences in the efficacy of safinamide in different ethnic groups has also been explored in a *post hoc* analysis of data from the SETTLE study which reported that it was similarly effective in improving daily ON-time without troublesome dyskinesia and motor function in both Asian and Caucasian PD patients (26).

### 3.3 Real-world data

Adjunctive safinamide treatment over 12 months has also been evaluated in the real-world setting in a large European observational study (SYNAPSES) of 1,610 PD patients from six countries who were experiencing wearing-off of their PD medication. The analysis found that the proportion of patients experiencing dyskinesia decreased from baseline (39.2%) to 12 months (27.8) (27), which supports the findings of long-term safinamide treatment in controlled trials.

### 3.4 Insights and knowledge gaps

Controlled clinical trials are recognized as the cornerstone of evaluating a drug’s efficacy and safety, but observational data from real-world studies are a helpful resource to help bridge the gap between controlled trials and clinical practice (28). Real-world data provides valuable information about the effectiveness of treatment outside of the controlled environment of a clinical trial, and importantly reflects the heterogenous PD population that clinicians are presented with daily that varies in age, underlying genetics, associated comorbidities and treatment history. Evaluation of the available data can also provide insights into the timeline of a drug’s clinical effects and potential mechanisms.

We know from the controlled trials of safinamide treatment that the reported reductions in dyskinesia have been relatively modest. However, these trials were powered primarily to assess the effects of safinamide on daily ON-time and OFF-time, and not specifically its effect on dyskinesia, which may explain in part why the reported effects of safinamide on dyskinesia are inconsistent across these studies. The overall findings from these studies suggest that safinamide treatment results in significant improvement in ON-time without troublesome dyskinesia relatively quickly, but there may be a small, transient increase or persistence of dyskinesia initially in a proportion of patients. This may improve with continued treatment, particularly in patients who have moderate-to-severe levodopa-induced dyskinesia at baseline. These observations are consistent with the dual-timeline hypothesis proposed in this article, whereby early improvements in motor ON-time reflect dopaminergic effects while later changes in dyskinesia may relate to modulation of glutamatergic pathways.

The effects of safinamide observed in the controlled trials described above are not always replicated in real-world clinical practice, particularly in the early weeks of treatment. There are several possible factors that contribute to this, most importantly that in real-world clinical practice safinamide is not always used as the first adjunctive therapy option, often being added to the PD treatment regimen after multiple other adjunctive therapies. As a result, in most patients the dopaminergic load will already be relatively high, and many will have substantial baseline dyskinesia when starting safinamide treatment. In addition, adjustments to the daily levodopa dose as part of therapy optimization can make it difficult to determine any specific impact of safinamide on dyskinesia.

## 4 The effects of safinamide on non-motor PD symptoms

PD is associated with a broad spectrum of NMS, and patients can also experience non-motor fluctuations, all of which can have a significant negative impact on their daily lives (3, 29–31). Effective management of NMS alongside motor symptoms is therefore important to ensure patients have the best possible quality of life. Since non-dopaminergic mechanisms often underpin NMS in PD, it is thought that the antiglutamatergic effects of safinamide may contribute to its beneficial effects on NMS in addition to its dopaminergic effects on motor ON time (32).

Safinamide treatment does appear to provide broader benefits on NMS than many other adjunctive therapies, possibly due to its dual mechanism of action. Published evidence supports improvements in apathy, mood/depression and pain with safinamide treatment, with additional benefits on sleep, fatigue, urinary symptoms and cognition (11, 32–37). In a prospective, open-label, single-arm study conducted in five centers in Spain of the effectiveness of safinamide on NMS in PD patients (SAFINONMOTOR), safinamide treatment over 6 months was found to improve overall NMS burden and quality of life in subjects who had a severe or very severe NMS burden at baseline (38).

## 5 A phenotype-based approach to selection of safinamide treatment

Safinamide is indicated as an add-on to oral levodopa therapy in patients with mid- to late-stage PD patients experiencing motor fluctuations. However, the observed effects on both motor symptoms and NMS from a range of controlled and observational clinical trials coupled with its known dual mechanism of action, suggest that adjunctive safinamide treatment may have a broader therapeutic range and be particularly beneficial for certain PD patient phenotypes (Table 1). Rather than specifying a fixed and specific profile of a candidate who may be suitable for safinamide treatment, it may be better viewed as a mechanistically distinct adjunctive option whose role can be tailored according to patient’s symptom profile, treatment history, and tolerability considerations. Further research is needed to better define phenotype-driven strategies for the optimal use of safinamide, but the following categories are suggested for consideration.

**Table 1:** Clinical phenotypes in Parkinson’s disease in which safinamide may be considered as adjunctive therapy based on its dual mechanism of action.

Clinical phenotype	Typical clinical scenario	Rationale for safinamide use
Fluctuation-dominant phenotype	Patients experiencing wearing-off despite optimized oral levodopa therapy	Potent reversible MAO-B inhibition enhances dopaminergic transmission and increases daily ON-time without troublesome dyskinesia. Particularly useful in patients at increased risk of hallucinations or neuropsychiatric adverse effects
Dyskinesia-prone phenotype	Patients experiencing wearing-off in whom further levodopa dose escalation may worsen dyskinesia	Safinamide increases ON-time without worsening troublesome dyskinesia. Modulation of abnormal glutamatergic transmission may contribute to stabilization of basal ganglia network activity and potential gradual improvement in dyskinesia.
Non-motor heavy phenotype	Patients with prominent non-motor symptoms such as pain, sleep disturbance, fatigue, or mood symptoms	Dual dopaminergic and antiglutamatergic mechanisms may contribute to improvements in selected non-motor symptoms, particularly pain, fatigue, and sleep disturbance.

MAO-B, monoamine oxidase type B.

### 5.1 Fluctuation-predominant phenotype with increased risk of neuropsychiatric adverse events

Clinical studies have demonstrated that adjunctive safinamide treatment has a rapid effect in PD patients experiencing motor fluctuations with their current oral treatment regimen, significantly increasing ON-time with no or non-troublesome dyskinesia and decreasing OFF time (14, 15, 20, 21). Safinamide is also generally well tolerated across age groups represented in these studies, although data in very elderly or frail populations is limited. Indeed, safinamide treatment is associated with a low rate of hallucinations even in older adults with PD; less than 3% of patients reported hallucinations in pivotal trials (14, 15, 20, 21). This rate is slightly lower than that reported for rasagiline, another MAO-B inhibitor, (~4%) (39, 40), and considerably lower than that observed with dopamine agonists such as pramipexole (10–17%) and ropinirole (6–12%) (41), making safinamide a favorable choice in patients who might be at risk. Safinamide is also reported to be well-tolerated in patients with cognitive vulnerability (27). Treatment decisions should therefore be individualized, taking into account the patient’s comorbidities, polypharmacy, and overall functional status.

Although safinamide has demonstrated a relatively favorable neuropsychiatric safety profile compared with dopamine agonists, it is important to recognize that most pivotal clinical trials excluded patients with advanced dementia or unstable psychiatric disease. Consequently, evidence regarding the safety and tolerability of safinamide in frail older adults with significant cognitive impairment remains limited. In routine clinical practice, careful monitoring for neuropsychiatric and autonomic adverse effects is therefore advisable, particularly in older patients with multimorbidity or complex polypharmacy.

### 5.2 Dyskinesia-prone phenotype

Patients with PD who are prone to levodopa-induced dyskinesia represent another clinical phenotype in whom safinamide may have particular therapeutic relevance. Clinically, a dyskinesia-prone phenotype may include patients with established levodopa-induced dyskinesia, a history of dyskinesia after levodopa dose escalation, a narrow therapeutic window between wearing-off and peak-dose dyskinesia, high cumulative dopaminergic load, or moderate-to-severe dyskinesia at baseline. Many adjunctive therapies used to manage motor fluctuations exert dopaminergic effects that may increase the risk of new-onset dyskinesia or exacerbate existing involuntary movements (42, 43). In clinical practice, this presents a therapeutic dilemma when patients experience wearing-off but further escalation of levodopa therapy would likely worsen dyskinesia.

Evidence from controlled clinical trials indicates that safinamide increases daily ON-time without troublesome dyskinesia when used as an adjunct to levodopa (14, 20). In the pivotal studies, improvements in motor ON-time were observed without a parallel increase in dyskinesia severity, suggesting that the drug enhances dopaminergic benefit while maintaining a relatively stable dyskinesia profile (23). However, these trials were not primarily designed to evaluate antidyskinetic effects, and the overall reductions in dyskinesia reported in controlled studies have generally been modest.

From a mechanistic perspective, the antiglutamatergic properties of safinamide may provide a biological rationale for potential benefits in dyskinesia-prone patients. Abnormal glutamatergic transmission within the basal ganglia has been implicated in the pathophysiology of levodopa-induced dyskinesia, contributing to maladaptive synaptic plasticity and excessive motor output (19, 44). By modulating glutamate release through state-dependent sodium channel blockade, safinamide may help stabilize these network processes and potentially attenuate dyskinesia over time (17).

Importantly, the clinical manifestations of this mechanism may not be immediate. While the dopaminergic effects of safinamide often produce rapid improvements in motor ON-time within the first weeks of treatment, modulation of glutamatergic pathways may occur more gradually. As discussed earlier, post-hoc analyses and observational studies suggest that dyskinesia may initially persist or even transiently increase before gradually improving with longer-term treatment. This temporal dissociation aligns with the dual-timeline hypothesis proposed in this article, whereby early dopaminergic benefits are followed by slower network-level modulation related to glutamate regulation.

From a practical perspective, safinamide may therefore represent a useful adjunctive option in patients with motor fluctuations in whom clinicians wish to improve OFF periods without substantially increasing dyskinesia burden. In these individuals, clinicians should interpret early dyskinesia changes cautiously and allow sufficient treatment duration before judging the overall therapeutic effect. This phenotype therefore exemplifies the potential temporal dissociation between early dopaminergic motor benefits and later glutamatergic modulation proposed in the dual-timeline framework.

### 5.3 Non-motor heavy phenotype

Data from controlled and observational clinical trials suggest that safinamide treatment has a broad beneficial effect on a wide range of NMS, including in patients with a high NMS burden at baseline (11, 32–37). It is thought that the effects of safinamide on NMS build gradually, are experience later than motor-ON effects, and are likely due to its antiglutamatergic properties. Studies have shown particular improvements in subgroups of patients experiencing pain (35, 45, 46) or fatigue/sleep disturbances (47).

While additional RCTs would be valuable, confirmation of the proposed dual timeline of effects will also require well-designed longitudinal, pragmatic real-world studies. Such studies should prospectively evaluate patients treated in routine clinical practice, where safinamide is often introduced at different stages of disease progression and in combination with varying background therapies. Pragmatic designs incorporating standardized assessments of dyskinesia, NMS, and levodopa dose adjustments over extended follow-up periods would provide a more ecologically valid understanding of treatment trajectories. In particular, repeated-measures analyses capturing early (weeks) versus later (months) outcomes could help clarify whether the apparent delayed benefits on dyskinesia and NMS represent a true mechanistic effect related to glutamate modulation or reflect confounding factors such as medication optimization or regression to the mean. Long-term observational cohorts with predefined evaluation timepoints would therefore be instrumental in substantiating the hypothesis of a dual timeline clinical response and in refining patient selection strategies.

In our view, patients most likely to benefit from this phenotype-oriented approach are those with motor fluctuations accompanied by prominent pain, sleep disturbance, fatigue, mood symptoms, apathy, or high overall NMS burden, particularly when these symptoms fluctuate with motor state; however, these potential benefits should be considered exploratory and secondary to safinamide’s established role in motor fluctuation management.

## 6 Clinical implications of a dual timeline effect of safinamide

The hypothesis that safinamide may exert a dual timeline of clinical effects, an early dopaminergic phase followed by a later glutamatergic-modulating phase, has potential implications for therapeutic positioning, patient counseling, and follow-up strategy in routine clinical practice (Figure 1).

### 6.1 Reframing safinamide beyond a conventional MAO-B inhibitor

In clinical practice, safinamide is often grouped with other MAO-B inhibitors and prescribed primarily for the management of motor fluctuations. While this remains its established indication (13–15), its additional glutamate-modulating properties suggest that its clinical effects may extend beyond dopaminergic enhancement alone. If safinamide produces rapid dopaminergic improvements in motor ON time followed by more gradual modulation of dyskinesia and selected NMS, clinicians may observe two distinct phases of therapeutic response rather than a single static treatment effect (Figure 1).

### 6.2 Practical implications for expectation setting

One important clinical implication of this concept relates to expectation setting at treatment initiation. Patients should be informed that improvements in motor ON time are typically observed within the first few weeks of therapy, reflecting the drug’s dopaminergic mechanism of action. In contrast, potential benefits on dyskinesia and certain NMSs, including pain, sleep disturbance, or fatigue, may emerge more gradually over several months (35, 38, 45, 46), if they occur at all. Without appropriate counseling, both clinicians and patients may prematurely judge treatment ineffective if later-phase benefits are not yet evident during early follow-up. Similarly, transient persistence or mild early increases in dyskinesia should not necessarily be interpreted as treatment failure, particularly when motor fluctuations have improved.

### 6.3 Structuring follow-up according to the dual timeline

Recognizing that there is potentially a dual timeline of clinical effects with safinamide may also help clinicians interpret treatment response more appropriately and structure follow-up assessments accordingly. An early review at approximately 4–6 weeks after treatment initiation allows evaluation of improvement in motor fluctuations, assessment of tolerability, and monitoring for neuropsychiatric adverse effects. A later review at approximately 3–6 months may provide a more appropriate time point to reassess dyskinesia severity and evaluate potential changes in non-motor symptoms such as pain, sleep disturbance, fatigue, or mood. The proposed 3–6 month reassessment interval should be viewed as a pragmatic clinical window rather than a proven mechanistic threshold. Early motor effects of safinamide are typically assessed within the first weeks of treatment, whereas dyskinesia and NMS outcomes have generally been evaluated at longer time points in clinical and observational studies, including 24-week/6-month NMS and pain assessments and longer-term dyskinesia analyses extending to 52 weeks or 24 months (23, 38). Thus, reassessment at 3–6 months provides a clinically practical interval to evaluate possible later-emerging changes while still allowing earlier discontinuation or adjustment in patients with poor tolerability or no motor benefit. The use of structured assessment tools, including patient motor diaries, DRS, or validated NMS scales, may facilitate objective longitudinal evaluation and help distinguish treatment-related effects from natural symptom variability (Table 2).

**Table 2:** Suggested clinical follow-up framework after initiation of safinamide based on the proposed dual timeline of pharmacological effects.

Treatment phase	Early phase (dopaminergic effects)	Later phase (glutamatergic modulation)
Pharmacological profile	Potent reversible MAO-B inhibition leading to enhanced dopaminergic transmission	Gradual modulation of abnormal glutamatergic activity through voltage-gated sodium channel blockade and reduction of stimulated glutamate release
Clinical effects	Rapid improvement in motor fluctuations and increased ON-time without troublesome dyskinesia	Potential gradual effects on dyskinesia and selected non-motor symptoms such as pain, sleep disturbance, or fatigue
Typical time course	Benefits typically apparent within the first weeks of treatment	Effects may emerge gradually over several months
Suggested follow-up strategy	Review at approximately 4–6 weeks to assess improvement in motor fluctuations, tolerability, and neuropsychiatric adverse effects	Reassessment at approximately 3–6 months to evaluate dyskinesia severity, non-motor symptom burden, and overall functional response

MAO-B, monoamine oxidase type B.

### 6.4 Implications for phenotype-oriented prescribing

The dual timeline framework may also support a more phenotype-oriented approach to treatment selection. In patients with fluctuation-predominant disease, the primary therapeutic objective may be rapid improvement in motor ON time. In contrast, in dyskinesia-prone or non-motor-heavy phenotypes, safinamide may be considered with the understanding that some of its potential benefits may emerge more gradually. This perspective encourages clinicians to interpret treatment response over an appropriate time horizon and avoids overly binary judgments based solely on short-term outcomes.

### 6.5 A temporally-informed treatment perspective

The dual timeline model proposed here remains conceptual and is derived from integration of pharmacological principles, clinical trial observations, and real-world clinical experience. Existing trials were not specifically designed to evaluate delayed antidyskinetic or non-motor effects, and factors such as levodopa dose adjustments or disease progression may influence longer-term outcomes. Nevertheless, considering the possibility of temporally distinct therapeutic phases may help clinicians align follow-up timing with pharmacological plausibility, interpret dyskinesia evolution more cautiously, and individualize treatment goals over time. Prospective longitudinal studies will be necessary to validate this hypothesis and clarify its implications for clinical practice.

Although the emphasis of this section is on therapeutic trajectory rather than contraindications, it remains important to note that safinamide should be avoided in severe hepatic impairment and in combination with other MAO inhibitors, and used with appropriate caution in patients with significant psychiatric instability or complex polypharmacy, in accordance with prescribing guidance (7, 8). These considerations, however, do not alter the central conceptual framework of a staged clinical response. Prospective studies with predefined longitudinal assessments will therefore be necessary to validate this temporal model.

### 6.6 Alternative explanations and limitations of the dual timeline model

The dual timeline model proposed here remains conceptual and hypothesis-generating. Existing RCTs were designed primarily to evaluate motor fluctuations rather than delayed antidyskinetic or non-motor effects, and post-hoc or observational findings cannot establish causality. Apparent gradual improvements in dyskinesia or NMS may reflect factors unrelated to safinamide’s glutamate-modulating properties, including levodopa dose adjustments, changes in concomitant therapy, regression to the mean, patient selection or attrition bias, adherence effects, natural symptom variability, or measurement variability in motor diaries and rating scales.

Accordingly, the proposed model should not be interpreted as evidence that safinamide has a proven delayed antidyskinetic effect. Rather, it provides a testable framework for future prospective studies. Ideally, such studies should include predefined early and later assessment points, stable or carefully documented levodopa dosing, standardized dyskinesia and NMS scales, patient motor diaries, and analysis of potential confounders. Where feasible, pharmacokinetic or biomarker measures may help clarify whether later clinical trajectories correlate with engagement of glutamatergic mechanisms.

## 7 Conclusion

The results of controlled clinical trials and real-world observational studies suggest that the therapeutic effects of safinamide when used as an add-on therapy to levodopa may have a dual timeline that likely results from its dual mechanism of action. While impairment of dopaminergic pathways is what commonly characterizes PD, the condition also affects other neurotransmitter systems (48). Therefore, it is plausible that PD medications that impact multiple pathways might have additional therapeutic benefits beyond the classical dopaminergic effect on motor ON-time (8).

The dopaminergic effects of safinamide, due to direct MAO-B inhibition, provide a rapid, noticeable improvement in daily motor ON-time without troublesome dyskinesia within the first weeks of treatment. However, there also appears to be a slower second-wave effect that builds gradually and is only observed after weeks or months of treatment, which may provide benefits in terms of improvements in dyskinesia, pain, and certain other NMSs. It can be hypothesized that these later-stage outcomes are due to glutamate modulating effects of safinamide and that slow recalibration of complex neural networks may be responsible for the observed clinical improvements in both motor and some NMSs over time with longer-term treatment. Safinamide therefore represents a pharmacologically distinct therapeutic option whose dual mechanism of action is reflected in a dual timeline of effect which warrants better and more refined clinical positioning. While current evidence supports its efficacy in motor fluctuations, further research is required to define its optimal place within phenotype-driven treatment algorithms.

## Data Availability

The original contributions presented in the study are included in the article/supplementary material, further inquiries can be directed to the corresponding author.
